# Planctomycetes dominate biofilms on surfaces of the kelp *Laminaria hyperborea*

**DOI:** 10.1186/1471-2180-10-261

**Published:** 2010-10-15

**Authors:** Mia M Bengtsson, Lise Øvreås

**Affiliations:** 1 Department of Biology, University of Bergen, Box 7803, N-5020 Bergen, Norway

## Abstract

**Background:**

Bacteria belonging to *Planctomycetes *display several unique morphological and genetic features and are found in a wide variety of habitats on earth. Their ecological roles in these habitats are still poorly understood. Planctomycetes have previously been detected throughout the year on surfaces of the kelp *Laminaria hyperborea *from southwestern Norway. We aimed to make a detailed investigation of the abundance and phylogenetic diversity of planctomycetes inhabiting these kelp surfaces.

**Results:**

Planctomycetes accounted for 51-53% of the bacterial biofilm cells in July and September and 24% in February according to fluorescence *in situ *hybridization (FISH) results. Several separate planctomycetes lineages within *Pirellulae*, *Planctomyces *and OM190 were represented in 16S rRNA gene clone libraries and the most abundant clones belonged to yet uncultured lineages. In contrast to the abundance, the diversity of the planctomycete populations increased from July to February and was probably influenced by the aging of the kelp tissue. One planctomycete strain that was closely related to *Rhodopirellula baltica *was isolated using selective cultivation techniques.

**Conclusions:**

Biofilms on surfaces of *L. hyperborea *display an even higher proportion of planctomycetes compared to other investigated planctomycete-rich habitats such as open water, sandy sediments and peat bogs. The findings agree well with the hypothesis of the role of planctomycetes as degraders of sulfated polymeric carbon in the marine environment as kelps produce such substances. In addition, the abundant planctomycete populations on kelp surfaces and in association with other eukaryotes suggest that coexistence with eukaryotes may be a key feature of many planctomycete lifestyles.

## Background

Bacteria belonging to the phylum *Planctomycetes *have revealed several remarkable features that set them apart from other bacteria. Their cryptic morphology led early microbiologists to mistake them for fungi, and the discovery of their cell compartmentalization, featuring membrane bounded organelles, raised fundamental questions about the evolution of eukaryotes [1,2]. Further, the unique anammox metabolism found in some planctomycetes has revolutionized the view of microbial nitrogen cycling [3]. The planctomycetes also possess cell walls without peptidoglycan, a characteristic that they share only with the obligate intracellular bacteria within *Chlamydiae*. In addition to the interest sparked by these unusual and fascinating features, planctomycetes have in later years attracted considerable attention because of their presence in a wide variety of environments on earth. By investigating bacterial communities using molecular methods (sequences coding for 16S rRNA), planctomycetes have been repeatedly detected in soil, sediments, marine and freshwater systems and in terrestrial hot springs to mention just a few (for a detailed review see [4]). However, their metabolic potential and function in these ecosystems is often unclear, as 16S rRNA gene sequence investigations only rarely give clues to ecological roles.

In the marine environment, DeLong and co-workers [5] found that planctomycetes were more abundant in clone libraries from marine aggregate (marine snow) attached bacteria than from free-living bacteria. Since then, results from several studies suggest that planctomycetes favor a biofilm lifestyle, adhering to surfaces in aquatic environments including marine sediments [6] (among others), diatom cells [7], seaweeds and other aquatic macrophytes [8,9]. *Rhodopirellula baltica *is an extensively studied marine particle-attached planctomycete. Its genome sequence reveals a large number of genes involved in the breakdown of sulfated polysaccharides [10], a carbon source found in marine photosynthetic organisms such as microalgae and seaweeds, who's detrital material is thought to generate marine snow. Such genes are also encountered in other planctomycete genomes and planctomycete-derived metagenomic fosmid libraries from seawater collected in upwelling zones [11]. The overrepresentation of such genes, and the association of *R. baltica *and other planctomycetes with marine snow has led to the hypothesis that heterotrophic planctomycetes are specialized degraders of sulfated polymeric carbon, for example in marine snow [10,11]. Given the significance of marine snow as part of the so-called "biological pump" of carbon in the oceans [12,13], planctomycetes may thereby be playing a crucial role in global carbon turnover [11]. Still, quantitative data on the distribution of planctomycetes in the marine environment and elsewhere is still scarce, and very little is known about the yet uncultured planctomycete lineages that are assumed to carry out the bulk of these globally critical processes.

Kelps are large brown seaweeds of the order *Laminariales*. They often form dense stands along rocky coastlines that are referred to as kelp forests. Kelp forest ecosystems are some of the most productive ecosystems in the world [14]. Their immense importance for coastal biodiversity, productivity and human economy has long been recognized in temperate regions of the world and is only beginning to be understood in the tropics [15]. Kelp forests along the Atlantic coasts of Europe are dominated by the large kelp *Laminaria hyperborea*. Bacteria associated to kelp are believed to be important in the carbon and nitrogen turnover in kelp forest food webs [16,17], but it is still not known what types of bacteria are involved in these processes. Recently, the seasonal dynamics of the cell density and bacterial community composition in biofilms on *L. hyperborea *were addressed. In this study, planctomycetes were frequently detected throughout the year but their abundance and phylogenetic relationships were not considered [18]. In order to address the importance of this group of bacteria in kelp forests, we therefore aimed to take an in-depth look at the abundance and phylogenetic diversity of planctomycete communities inhabiting *L. hyperborea *surface biofilms. This was achieved by using fluorescence *in situ *hybridization (FISH) to quantify their abundance and visualize their distribution in the biofilm as well as 16S rRNA gene clone library construction to elucidate their phylogenetic relationships, community composition and diversity at different times of the year. In addition, a selective cultivation approach was used to assess the culturability of planctomycetes from kelp surfaces.

## Results

### Abundance of planctomycetes in kelp surface biofilms

**Figure 1:**
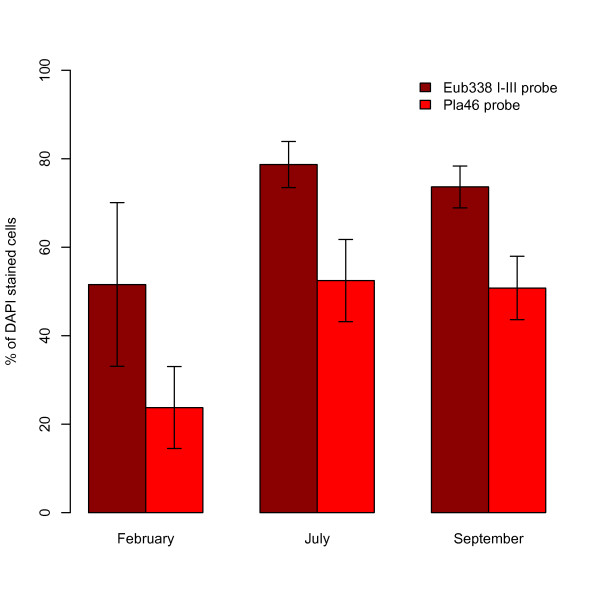
**Abundance of planctomycetes in kelp surface biofilms**. The abundance of cells stained by the *Planctomycetes *specific probe Pla46 and the general bacterial probe Eub338 I-III at three different sampling times as a percentage of total cells (DAPI stained). The height of the bars represents the average percentage values of six individual kelp plants sampled at each sampling occasion. Error bars indicate one standard deviation (± 1SD).

Quantification of planctomycetes in samples from July 2007, February 2007 and September 2008 using FISH showed that they make up a large part of the kelp surface biofilm community in all three sampling occasions. In July and September they dominated the community, with cells hybridizing with the *Planctomycetes*-specific probe Pla46 [19] accounting for over 50% of the total DAPI stained cells on average (Table 1 and Figure 1). In February, the planctomycetes were less abundant; with Pla46 hybridized cells corresponding to an average of 24% of total DAPI stained cells. Samples that were also subjected to hybridization with the Pir1223 [20] probe showed similar percentages (±1%) of hybridized cells as the with Pla46 probe (results not shown). Inspection of the cloned 16S rRNA gene sequences revealed that the Pir1223 target sequence was present in all clones except those belonging to the OM190 lineage (see the following sections) suggesting that the specificity of this probe needs to be reevaluated. The different formamide concentrations (20-40%) used in hybridization with the Pla46 probe did not change the proportion of Pla46 hybridized cells significantly (results not shown). The average proportion of the DAPI stained cells that hybridized with the Eub338 probes was 79% in July, 74% in September and 52% in February (Table 1 and Figure 1).

**Table 1:** A summary of the results

Sampling time	Avg. cells/cm^2 ^(DAPI) ± 1SD	Avg.% Eub338 I-III of DAPI ± 1SD	Avg.% Pla46 of DAPI ± 1SD	% Pla46 of Eub338 I-III	No. of clones	No. of OTUs (98%)	Shannon diversity index	Chao1 OTU richness estimate ± SE
February 2007	8.2e+06 ± 1.9e+06	51.6 ± 18.5	23.7 ± 9.3	45.9	73	20	2.56	29 ± 12.5

July 2007	7.4e+06 ± 4.8e+06	78.7 ± 5.2	52.5 ± 9.3	66.7	89	9	1.85	9 ± 0.73

September 2008	1.7e+07 ± 6.4e+06	73.6 ± 4.7	50.8 ± 7.2	69.0	89	15	2.32	16 ± 3.4

### Cell distribution of planctomycetes in the biofilms

**Figure 2:**
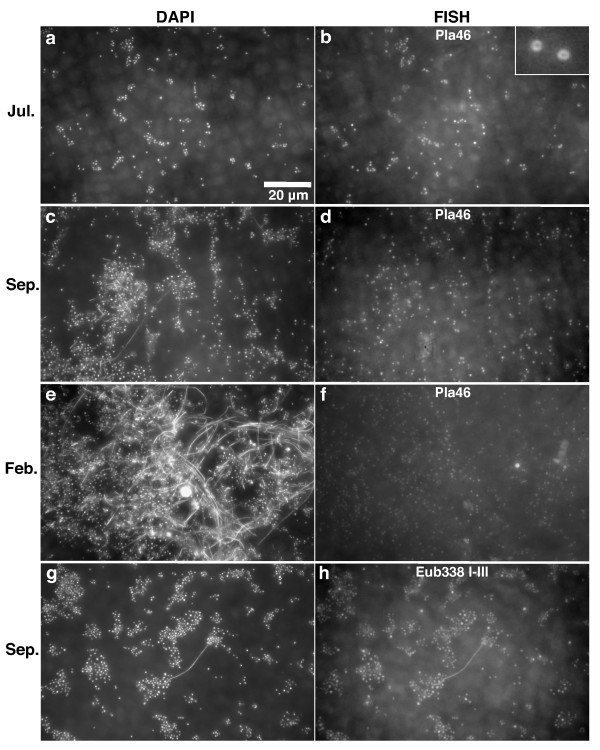
**Distribution of planctomycete cells in the biofilm**. Fluorescence microscopy images of *Laminaria hyperborea *surface biofilm. Images a, c, e and g show DAPI stained biofilm while b, d, f and h show FISH signals in the same microscope fields from hybridizations with either the Pla46 probe (b, d and f) or the Eub 338 I-III probe mix (h). Images show representative microscope fields of samples from July 2007 (a-b), September 2008 (c-d, g-h) and February 2007 (e-f). The enlarged inset image in b shows the typical ring shaped FISH signals of planctomycetes.

Fluorescence microscopy images of DAPI and FISH stained biofilm cells revealed a complex and variable microscopic landscape. The microbial cells appeared to be unevenly distributed on the kelp surface, often occurring in clusters (Figure 2a, c and 2g) or growing along straight lines (Figure 2c). Planctomycetes cells were found within all these structures, and appeared to grow evenly intermingled with other cells (Figure 2b, d and 2f). Fluorescence microscope images showed DAPI and FISH signals corresponding to different cell morphologies in the biofilm, ranging from long filaments, cocci of different sizes and small rods (Figure 2). The planctomycete FISH signals were always in the shape of small and medium sized cocci (Figure 2b, d and 2f) and displayed the "ring" shape typical of planctomycete cell organization [19] (Figure 2b inset). The Eub338 FISH signals included the whole range of morphologies (Figure 2h) and were both ring-shaped and solid.

### Isolation and cultivation of planctomycetes from kelp surfaces

**Figure 3:**
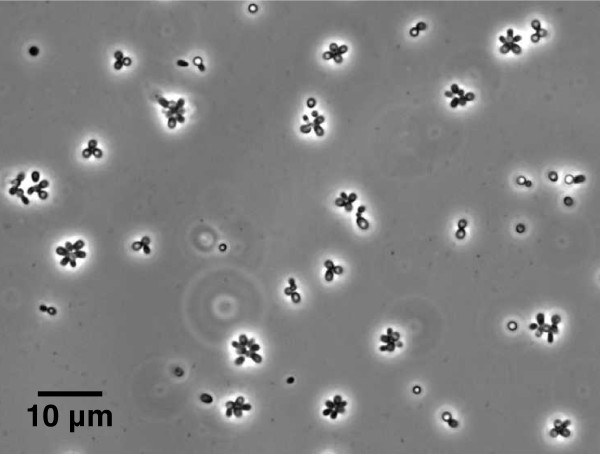
**The P1 strain**. A phase contrast photomicrograph showing the *Rhodopirellula *sp. strain P1 isolated from kelp surface biofilm, displaying ovoid cells, budding and rosette formation.

**Figure 4:**
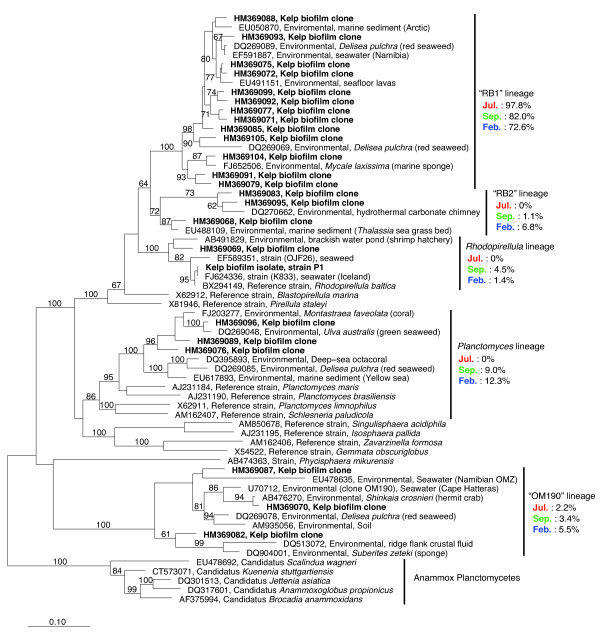
**Phylogenetic relationships of planctomycetes**. A maximum likelihood (PhyML) tree based on 16S sequences of *Planctomycetes*. An outgroup consisting of reference sequences from the *Verrucomicrobia *were used for tree calculation, but is not displayed in the tree. Bold letters designate sequences derived from the present study, which include one representative of each OTU and the P1 isolate. Reference sequences from the SILVA database are described by their GenBank accession numbers, origin of the sequence (environmental or cultured strain) and the habitat they were obtained from. The vertical lines mark phylogenetic lineages of interest. The percentage of each clone library that was made up of sequences from each phylogenetic lineage is indicated. Bootstrap values >60 (based on 1000 bootstraps) are displayed. The scale bar indicates 0.10 (10%) sequence divergence.

One strain, named "P1", belonging to *Planctomycetes *was isolated from kelp surface biofilm material from September 2008. It displayed morphological features typical for *Rhodopirellula baltica*, with ovoid cells and rosette formation (Figure 3). It formed pink colonies on M30 solid media that were visible after approximately seven days of incubation in room temperature after inoculation. It was closely related to the type strain of *Rhodopirellula baltica *(Figure 4, 99.5% 16S rRNA gene sequence similarity) and to *Rhodopirellula *strain K833 isolated from seawater in Iceland [21] (Figure 4, 99.9% sequence similarity). However, it was not closely related to any of the clone library sequences from kelp surface biofilms (Figure 4).

### Phylogenetic diversity of planctomycetes from kelp surface biofilms

Three clone libraries, from February 2007, July 2007 and September 2008, constructed with the *Planctomycetes*-specific primer Pla46f and the general bacterial primer 1542r were analyzed to gain insight into the phylogenetic diversity of the planctomycetes growing in kelp surface biofilms. In total, 266 clones were sequenced in the forward direction from the three clone libraries, resulting in partial 16S rRNA gene sequences of approximately 850 basepairs. Of these, only 9 sequences (3.4%) did not classify as belonging to *Planctomycetes *and were discarded from the further analyses. These unspecific sequences classified as *Deltaproteobacteria *(three), *Gammaproteobacteria *(two), *Actinobacteria *(two) and *Verrucomicrobia *(one) while one remained unclassified using the Greengenes G2Chip classifier [22]. The remaining 257 partial planctomycete 16S rRNA gene sequences clustered into 23 OTUs at 98% sequence similarity. Other OTU definitions (95-99%) gave different numbers of OTUs, but the general trends observed in the dataset were the same. One to six representative clones of each OTU were selected for sequencing in the reverse direction in order to assemble near full-length 16S rRNA gene sequences. Of the assembled sequences, three were removed from the analyses because of poor sequence quality and two because of indications of chimeric origin. The remaining 46 near full-length planctomycete 16S rRNA gene sequences have been deposited to GenBank under the accession numbers HM369064 to HM369109, and the sequence of the P1 isolate under HM369063.

**Figure 5:**
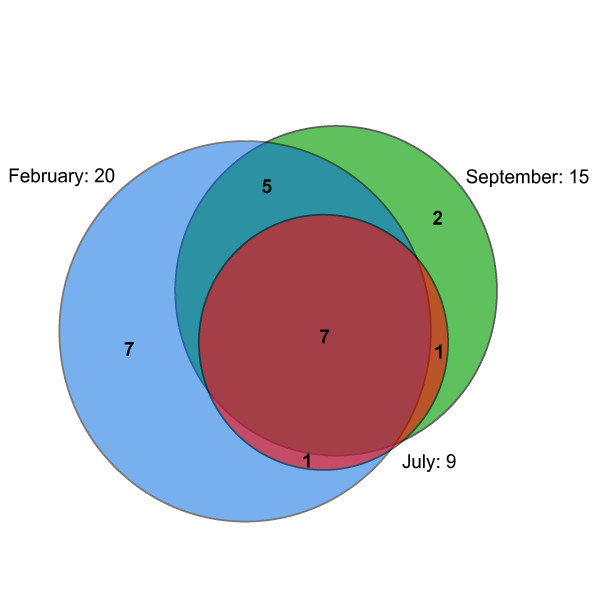
**Overlap of planctomycete OTUs between sampling times**. A Venn diagram describing the degree of OTU overlap between the different clone libraries. The total number of OTUs in each library is displayed outside the circles and the number of overlapping OTUs is given inside the areas of the circles. The area-proportional Venn diagram was generated at http://www.venndiagram.tk.

**Figure 6:**
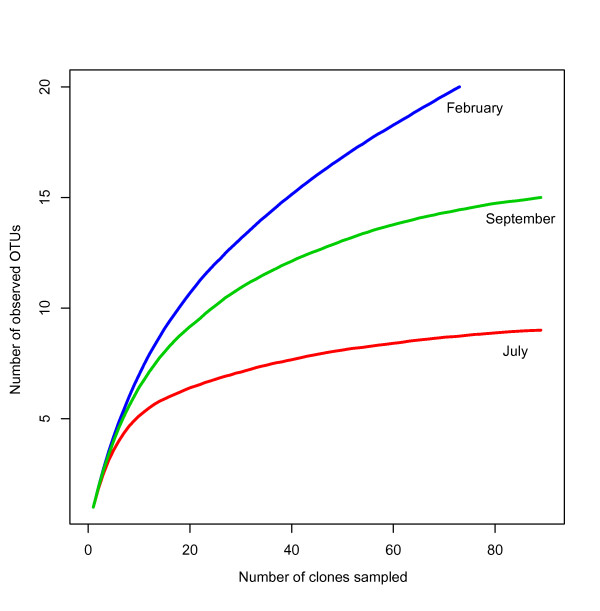
**OTU diversity of planctomycetes**. Rarefaction curves indicating the expected OTU richness of the clone libraries with different sampling efforts.

The clone libraries from February, July and September showed considerable overlap in OTU composition (Figure 5). The July library had the lowest OTU richness and consisted of a subset of the OTUs detected in the other two libraries. The highest OTU richness and the most unique OTUs (seven) were found in February. September was intermediate in OTU richness and the number of unique OTUs (Figs. 5 and 6). The diversity of the three clone libraries is illustrated in Figure 6 using rarefaction curves showing the expected number of OTUs encountered with clone sampling effort. July displays a near asymptotic curve, indicating low diversity, while September is intermediate and February displays the highest diversity. The Shannon diversity index and the Chao1 richness estimates for the clone libraries (Table 1) show the same relative diversity pattern.

The phylogenetic analysis of the near full-length sequences obtained in this study and other planctomycete sequences obtained from the Silva reference database [23] revealed that highly divergent lineages of the *Planctomycetes *phylum are represented in kelp surface biofilms (Figure 4). The kelp surface biofilm clone sequences appear to cluster within five major lineages that have been labeled as: "RB1" and "RB2" (defined in this study), *Rhodopirellula*, *Planctomyces *and "OM190". The "RB1" and "RB2" lineages appear more closely related to the *Rhodopirellula *and *Blastopirellula *genera than to the *Pirellula *genus and were given their labels based on that (RB = *Rhodopirellula*/*Blastopirellula*). Yet the phylogenetic analyses do not place them consistently with either of the genera. Sequence similarities of 86-90% to *Rhodopirellula baltica *and *Blastopirellula marina *indicate that they probably represent distinct phylogenetic lineages that could correspond to new genera according to conventional taxonomical practice. The "RB1" lineage was by far the most represented in all three clone libraries (Figure 4). Sequences that cluster within the "RB2", *Rhodopirellula *and *Planctomyces *lineages were only represented in September and February, indicating a seasonal difference, while OM190 representatives were present at low numbers in all three clone libraries (Figure 4).

## Discussion

To our knowledge, the kelp surface biofilms investigated in this study display the highest proportion of bacteria belonging to *Planctomycetes *reported in a natural bacterial community so far. This observation is consistent with earlier results from a DGGE based study on seasonal variation of *Laminaria hyperborea *(kelp) surface biofilm communities [18]. Other habitats where a high abundance of planctomycetes has been reported include seawater during a diatom bloom where planctomycetes related to *Pirellula *were detected attached to diatom cells and were among the dominant lineages in the bloom samples [7]. In investigations of sandy sediments containing algal cells [24,25], planctomycetes were also abundant, accounting for up to 20% of total cells, accompanied by *Cytophaga/Flavobacteria*. Gade and co-workers [20] used order-, genus- and strain specific FISH probes to detect planctomycetes in a range of aquatic habitats and recorded abundances up to 11% of total cells in some lakes. Peat bogs with *Sphagnum *moss have also been reported to harbor abundant (up to 13% of total bacterial numbers) planctomycete populations [26]. Similarly to kelp surfaces, these environments are all highly influenced by photosynthetic eukaryotes.

The studies mentioned above have all quantified planctomycetes using specific FISH probes. Several other studies have detected planctomycetes using PCR based cloning and fingerprinting methods followed by sequencing. Two of the most frequently used general bacterial PCR primers, targeting the 16S rRNA gene around *E. coli *positions 8-27 and 338-355, contain mismatches against planctomycete sequences [27,28]. This may have caused planctomycete abundances to be underestimated in many habitats, leading investigators to turn their attention towards bacterial groups that appear more abundant. Despite awareness of this problem, the literature and the sequence databases probably reflect a tradition of neglect towards the planctomycetes. In the light of this, it is difficult to say whether the dominance of planctomycetes on *Laminaria hyperborea *surface biofilms represents a unique feature of this habitat, or if other planctomycete-dominated bacterial communities have been overlooked until now. For example, Staufenberger and co-workers [29] did not detect planctomycetes in surface biofilms of another species of kelp (*Saccharina latissima*) using general bacterial primers for cloning and DGGE analysis. Yet, use of different primers has let to the detection of planctomycetes on both the kelps *S. latissima *and *Laminaria digitata *(Bengtsson, unpublished results).

A possible explanation for the suitability of kelp as a habitat for planctomycetes is its content of sulfated polysaccharides, a class of molecules that some marine planctomycetes are known for being able to degrade [10]. For example, *Laminaria hyperborea *contains fucoidan, a class of complex brown algal sulfated polysaccharides. These substances are secreted to the surface of *L. hyperborea *via mucilage channels [30]. It is reasonable to assume that planctomycetes living on kelp surfaces utilize substances produced by the kelp, for example fucoidan, as carbon sources. However, the presence of suitable carbon sources appears insufficient to explain the observed dominance of planctomycetes, as they must not only be able to grow and divide, but also outcompete other bacteria to be successful. Another contributing factor to the success of planctomycetes on kelp surfaces may be resistance to chemical antimicrobial defense compounds produced by the kelp. Antibacterial activity has been detected in extracts from many species of kelp, yet the substances responsible for the activity have often not been identified [31]. The lack of peptidoglycan in planctomycete cell walls makes them resistant to conventional cell wall targeting antibiotics like ampicillin. Resistance to other antibiotics, targeting for example protein synthesis (streptomycin) has also been reported in some marine planctomycetes [32,33].

In many cases the reference sequences that are the most closely related to kelp surface planctomycetes are obtained from other marine eukaryotes such as for example red and green seaweeds, corals, crustaceans and sponges (Figure 4). The frequent association of planctomycetes to eukaryotes has previously been noted [34]. This could point to a general lifestyle pattern of heterotrophic planctomycetes. They may combine an affinity for sulfated polysaccharides and other polymeric carbon molecules [10,11] produced by their eukaryote hosts with a resistance to eukaryote chemical defense molecules. The resulting competitive advantage over other bacterial groups that are utilizing the same kind of substrates, for example the *Bacteroidetes *[35] might be one of the keys to the success of planctomycetes in a wide variety of environments on earth.

Our results show differences between the different sampling times (February, July and September), in planctomycete abundance, OTU composition and diversity. For example, in February there is a relatively low abundance of planctomycetes (Figure 1) compared to July and September. This may be linked to the age of the kelp tissue, as the kelp lamina is older in February compared to in July and September due to the seasonal growth cycle of the kelp. Aging of the kelp tissue could be associated with lowered antibacterial chemical defense by the kelp, as the old kelp lamina is to be shed soon after February, and does therefore not need to be defended against microbial colonization. Without the presence of chemical defense substances, the planctomycetes could loose their competitive advantage over other bacterial groups, explaining their lower abundance in February. The senescence of the kelp tissue as it ages could also cause the appearance of new niches involved in degradation of different kelp constituents, thereby enabling the more diverse planctomycete communities that are observed in February compared to July and September (Table 1, Figure 6).

Among the different planctomycete lineages that are represented on the kelp, the lineage defined as "RB1" in this study appears to be the most abundant, accounting for a majority of the clones at all sampling times (Figure 4). The high abundance of RB1 planctomycetes may thus be the cause of the observed dominance of planctomycetes on kelp surfaces (Figs. 1 and 2). Their high abundance implies a lifestyle that makes them particularly successful on kelp surfaces. Yet the lineage also includes reference sequences from a variety of other marine habitats, indicating that RB1 is not a kelp-specific lineage. The RB1 and RB2 lineages, defined in this study, are clearly related to the "*Pirellulae*", a lineage including the genera *Pirellula*, *Rhodopirellula *and *Blastopirellula *(formerly all included in the genus *Pirellula*). Yet our phylogenetic analyses did not place them reliably with any of the described genera, indicated by the bootstrap support for the relevant branches in Figure 4. There are no sequences of cultured strains within the RB1 and RB2 lineages available in the databases. Another uncultured lineage, the so-called OM190 planctomycetes (Silva taxonomy) is also represented by clones from kelp surfaces at all sampling times, yet in low numbers. This is a lineage that appears to branch off deeply in the planctomycete tree and representatives have been detected in a variety of environments including seawater [36], soil [37] and marine eukaryotes [8,38]. These lineages have yet to be cultured and described and will reveal valuable information on planctomycete metabolism and evolution if cultivation is successful.

Using conventional approaches, the *Rhodopirellula sp*. strain P1 could easily be isolated. Several closely related strains have been brought into culture earlier [21]. However, the 16S rRNA gene sequence of P1 does not correspond to any of the abundant OTUs detected on the kelp surfaces, for example within the RB1 lineage. Kelp surfaces are nevertheless a promising source for isolation of novel planctomycete strains, using more ambitious and creative approaches that take into account the environmental factors experienced by bacteria on kelp surfaces. The rewards awaiting such attempts can be substantial, given the representation of highly divergent lineages of the planctomycete tree in kelp surface biofilms.

## Conclusions

Kelp (*Laminaria hyperborea*) surface biofilms have a uniquely high relative abundance of planctomycetes. Several distinct lineages are represented, and the diversity and composition of the planctomycetes change during the year, probably influenced by aging of the kelp tissue. The finding of abundant planctomycete populations in kelp surface biofilms agrees well with the view of heterotrophic planctomycetes as surface attached, specialized degraders of sulfated polysaccharides in the marine environment, as kelps are known to produce such substances. Furthermore, we wish to extend this view by hypothesizing that many heterotrophic planctomycetes share a preference of intimate coexistence with eukaryotes, which may be linked to antibiotic resistance. The study addresses the urgent need for more detailed, quantitative knowledge on the diverse marine planctomycetes.

## Methods

### Sample collection and preparation

Kelp (*Laminaria hyperborea*) was collected at one site near Bergen, Norway (60° 09.706' N, 5° 02.371' E) in February 2007 and in July 2007. These sampling times were selected based on a previous study that detected low (February) and high proportions (July) of planctomycetes at these times [18]. In addition, kelp was sampled at the same site in September 2008 to obtain fresh biofilm material for cultivation of planctomycetes. Six replicate kelp individuals were collected from a depth of 5 to 9 m by dredging from a boat at each sampling occasion and were kept cool until further processing (a few hours). Biofilm samples were obtained from the middle part of the kelp lamina (blade) of each kelp individual. The lamina areas used for biofilm sampling were thoroughly washed with sterile seawater. Biofilm for DNA extraction was sampled by scraping off material from the kelp surface with a sterile scalpel as described previously [18]. Biofilm for FISH was sampled by cutting out whole pieces of the kelp lamina in order for the attached biofilm to remain intact. Sample collection and preparation procedures are described in greater detail in [18].

### FISH

Kelp lamina pieces (1 × 0.5 cm) were fixed in 2% buffered paraformaldehyde overnight, washed twice in 50% EtOH in PBS and stored in the same solution at -20°C. Prior to FISH, the kelp pieces were dehydrated in 96% EtOH and air-dried. Each sample kelp piece was further divided into 0.5 × 0.5 cm pieces, that were used for hybridization either with the general Bacterial probe mix Eub338 I-III [28] or the planctomycete specific probe Pla46 [19]. In addition, a subset of samples were hybridized with the probe Pir1223 [20] that is reported to be specific for the genera *Pirellula*, *Blastopirellula *and *Rhodopirellula *(formerly all included in *Pirellula*). Several samples were also hybridized with the Non338 probe to check for signals caused by unspecific hybridization or autofluorescence of bacterial cells. All probes were bound to the fluorochrome Cy3, as previous investigations have shown that it gives superior fluorescence signals over the otherwise troublesome autofluorescence of the kelp cells compared to other fluorochromes such as fluorescein (Bengtsson, unpublished data). The formamide concentrations in the hybridization solution for the respective probes were 35% for the Eub338 I-III mix, 30% for Pla46 and 30% for Pir1223. Formamide concentrations of 20, 25, 30, 35 and 40% were evaluated on a subset of the September samples for the Pla46 probe. FISH was carried out according to [39] with some modifications. In summary, the dry kelp pieces were soaked in hybridization solution and hybridized at 46°C for 3 hours inside capped 0.5 ml plastic tubes. After stringent washing and subsequent washing with dH2O, the kelp pieces were counter-stained with DAPI and mounted on glass slides as described in [18].

### Fluorescence microscopy

Digital images of randomly selected microscopic fields were captured for counting of DAPI stained cells and FISH hybridized cells. Image capture and counting were carried out as previously described [18]. The percentage FISH hybridized cells of the total cell count (DAPI stained cells) was calculated for every individual microscope field captured, and an average percentage was calculated for each sample.

### Isolation and cultivation of planctomycetes from kelp surfaces

Freshly scraped off biofilm material from September 2008 suspended in sterile seawater was used to inoculate M30 medium [4] diluted in 3/4 parts sterile seawater supplemented with ampicillin (0.2 mg/ml). After growth was detected, the liquid culture was plated out on M30 medium solidified with gellan gum (Gelzan, Sigma-Aldrich), and individual colonies were picked and re-plated several times to obtain pure cultures.

### DNA extraction

Scraped off biofilm was suspended in sterile filtered and autoclaved seawater and the cells were pelleted by centrifugation. DNA was extracted from the pellets as previously described [18]. Equal volumes of the DNA extracts from the 6 replicate kelp plants from each sampling time were pooled into one sample per sampling time. The purpose if this was to obtain an overall picture of the planctomycete populations at each sampling time. Variation in OTU composition between individual kelp laminae is not captured by this approach, but has been addressed previously for the whole bacterial communities [18]. The pooled DNA extracts (from February 2007, July 2007 and September 2008) were used for the subsequent PCR amplification and clone library construction.

### PCR amplification and clone library construction

The *Planctomycetes *specific forward primer Pla46f (5'-GGA TTA GGC ATG CAA GTC-3') complementary to the Pla46 FISH probe [19] and the general bacterial reverse primer 1542r (5'-AAG GAG GTG ATC CAG CCG CA-3') [40] were used to amplify a near full length fragment of the 16S rRNA gene of *Planctomycetes*. PCR conditions were: 94°C for 5 min, 25 cycles of 94°C for 1 min, 60°C for 1 min, 72°C for 2 min, and final elongation at 72°C for 10 min. Each 25 μl PCR reaction contained nuclease-free water, F511 buffer (Finnzymes), 0.1 mM of each dNTP (F506L, Finnzymes), 0.02% BSA, 0.5 μM of each primer, 0.02 U Dynazyme II F501-L (Finnzymes), and approximately 30 ng template DNA. Three clone libraries, one from each sampling occasion, were constructed using the TOPO TA cloning kit (Invitrogen). Ninety-six clones were picked from each clone library. Cloned fragments were reamplified using the supplied M13 primer pair according to the manufacturers instructions.

### Sequencing and sequence processing

All cloned fragments were sequenced in one direction using the Pla46f primer. Sequencing was carried out with the BigDye Terminator v3.1 kit (Applied Biosystems) at the Bergen Sequencing Facility http://www.seqlab.uib.no using an ABI 3700 sequencing system. Base calling from the chromatogram files was done using the Phred software [41] (version 0.020425.c). The resulting sequences representing partial fragments of the 16S rRNA gene were used to select a subset of clones to sequence in the reverse direction in order to obtain near complete length 16S rRNA gene fragments. The sequences were trimmed to approximately 750 bp of good quality sequence and aligned against the Silva seed alignment (release 102) using the SINA web aligner [23]. The alignment was imported into the ARB software package [42] (version 5.0) and was manually edited to improve alignment quality. The resulting alignment was used to create a distance matrix in ARB, which was used to cluster the sequences into OTUs using the furthest neighbor algorithm in the Mothur software [43] (version 1.9.0). Rarefaction and overlap analysis were carried out in Mothur. The Shannon diversity index and the Chao1 richness estimate was calculated in the R statistical environment ([44], functions: diversity and estimateR, package: vegan). Based on the OTU clustering, one to six representatives of each OTU were sequenced in reverse using the 1542r primer. The resulting sequences were assembled using CAP3 [45] with the corresponding forward sequences to build contigs spanning nearly the full length of the 16S rRNA gene. The contigs were manually cropped to roughly the same length using the Phred base quality scores of the ends of the contigs as a guide. The resulting same-length (about 1250 bp), good quality contiguous sequences were checked for chimeras using Bellerophon [46] through the online Greengenes interface [22]. The *Rhodopirellula sp*. strain P1 was sequenced in forward and reverse direction several times with different 16S rRNA gene primers. The individual sequence reads were manually assembled into one full-length consensus sequence.

### Phylogenetic tree reconstruction

The near full-length sequences were aligned using the SINA web aligner, imported into ARB and edited as described in the previous section. Reference sequences that were closely related to the clone sequences from this study and sequences from cultured planctomycetes were selected from the SILVA database and were included in the tree calculations. Several tree calculation methods including neighbor joining (NJ), maximum likelihood (ML) and maximum parsimony (MP) were used in combination with different conservatory filters in ARB and the tree topologies compared to ensure a reliable result. The final ML tree was calculated in ARB with 175 sequences using PhyML [47] applying bootstrap analysis (1000 bootstraps) and no filter. Four *Verrucomicrobia *sequences (accession numbers: AY271254, DQ302104, AB297805, AB297806) were used as an outgroup in the tree calculation. The tree was edited by removing some of the reference sequences for clarity of presentation and the final result is shown in Figure 4.

## Authors' contributions

MMB carried out the sampling, laboratory work and data analysis and wrote the manuscript. LØ conceived the study, supervised the laboratory work and data analysis and participated in editing the manuscript.
